# Supplementary material for: Neoadjuvant Chemotherapy Followed by Concurrent Chemoradiation Versus Adjuvant Chemotherapy Following Concurrent Chemoradiation for Locally Advanced Cervical Cancer: A Network Meta-Analysis
Source: Cancers (Basel). 2025 Jan 11;17(2):223. doi: 10.3390/cancers17020223 (PMC11764324; doi:10.3390/cancers17020223)
Supplement: Supplementary file 1 [file cancers-17-00223-s001.zip › Table S1.pdf]

## Supplemental Table S1. Search strategy.

**Pubmed**

| # | **Search terms** |
|---|---|
| 1 | cervical cancer AND (chemoradiotherapy OR chemoradiation OR radiochemotherapy) AND (neoadjuvant chemotherapy OR adjuvant chemotherapy) AND randomized trial |

**Medline**

| # | **Search terms** |
|---|---|
| 1 | cervical cancer AND (chemoradiotherapy OR chemoradiation OR radiochemotherapy) AND (neoadjuvant chemotherapy OR adjuvant chemotherapy) AND randomized trial |

**Embase**

| # | **Search terms** |
|---|---|
| 1 | cervical cancer AND (chemoradiotherapy OR chemoradiation OR radiochemotherapy) AND (neoadjuvant chemotherapy OR adjuvant chemotherapy) AND randomized trial |
