# Supplementary material for: Neoadjuvant Chemotherapy Followed by Concurrent Chemoradiation Versus Adjuvant Chemotherapy Following Concurrent Chemoradiation for Locally Advanced Cervical Cancer: A Network Meta-Analysis
Source: Cancers (Basel). 2025 Jan 11;17(2):223. doi: 10.3390/cancers17020223 (PMC11764324; doi:10.3390/cancers17020223)
Supplement: Supplementary file 1 [file cancers-17-00223-s001.zip › Table S2.pdf]

Supplemental Table S2. Assessments of the risk of bias.

Study	Random sequence generation (selection bias)	Allocation concealment (selection bias)	Blinding of the participants and personnel (performance bias)	Blinding of outcome assessment (detection bias)	Incomplete outcome data (attrition bias)	Selective reporting (reporting bias)	Other bias
da Costa SCS et al. (2019) [12], CIRCE							
Authors' judgement	Low risk	Low risk	High risk	Unclear risk	Low risk	Low risk	Low risk
Support for judgement	Adequate method for randomization	Adequate allocation concealment	No blinding of the participants and personnel	Unclear blinding for outcome assessors	No incomplete outcome data	Report of all outcomes	No additional bias
McCormack M et al. (2023) [13], INTERLACE							
Authors' judgement	Unclear risk	Unclear risk	Unclear risk	Unclear risk	Unclear risk	Unclear risk	Unclear risk
Support for judgement	Unclear method for randomization	Unclear allocation concealment	Unclear blinding of the participants and personnel	Unclear blinding for outcome assessors	Unclear incomplete outcome data	Unclear report of all outcomes	Unclear additional bias
Li F et al. (2024) [14]							
Authors' judgement	Unclear risk	Unclear risk	High risk	Unclear risk	Low risk	Low risk	Low risk
Support for judgement	Unclear method for randomization	Unclear allocation concealment	No blinding of the participants and personnel	Unclear blinding for outcome assessors	No incomplete outcome data	Report of all outcomes	No additional bias
Dueñas-González A et al. (2011) [8]							
Authors' judgement	Low risk	Low risk	High risk	Unclear risk	Low risk	Low risk	Low risk
Support for judgement	Adequate method for randomization	Adequate allocation concealment	No blinding of the participants and personnel	Unclear blinding for outcome assessors	No incomplete outcome data	Report of all outcomes	No additional bias
Tovanabutra C et al. (2021) [10], ACTLACC							
Authors' judgement	Low risk	Low risk	Unclear risk	Low risk	Low risk	Low risk	Low risk
Support for judgement	Adequate method for randomization	Adequate allocation concealment	Unclear blinding of the participants and personnel	Adequate blinding for outcome assessors	No incomplete outcome data	Report of all outcomes	No additional bias
Mileshkin LR et al. (2023) [11], OUTBACK							
Authors' judgement	Low risk	Low risk	High risk	Low risk	Low risk	Low risk	Low risk
Support for judgement	Adequate method for randomization	Adequate allocation concealment	No blinding of the participants and personnel	Adequate blinding for outcome assessors	No incomplete outcome data	Report of all outcomes	No additional bias
